# *In Vitro* Comparison of the Activity Requirements and Substrate Specificity of Human and *Triboleum castaneum* PINK1 Orthologues

**DOI:** 10.1371/journal.pone.0146083

**Published:** 2016-01-19

**Authors:** Liesbeth Aerts, Katleen Craessaerts, Bart De Strooper, Vanessa A. Morais

**Affiliations:** 1 Center for the Biology of Disease, Flemish Institute for Biotechnology (VIB), Leuven, Belgium; 2 Center for Human Genetics, Leuven Institute for Neurodegenerative Disorders and University Hospitals Leuven, University of Leuven, Leuven, Belgium; 3 Institute of Neurology, University College London, London, United Kingdom; 4 Instituto de Medicina Molecular, Faculdade de Medicina, Universidade de Lisboa, Lisbon, Portugal; University of Padova, ITALY

## Abstract

Mutations in the gene encoding the mitochondrial kinase PINK1 cause early-onset familial Parkinson’s disease. To understand the biological function of PINK1 and its role in the pathogenesis of Parkinson’s disease, it is useful to study its kinase activity towards substrates both *in vivo* and *in vitro*. For *in vitro* kinase assays, a purified *Triboleum castaneum* PINK1 insect orthologue is often employed, because it displays higher levels of activity when compared to human PINK1. We show, however, that the activity requirements, and more importantly the substrate specificity, differ between both orthologues. While *Triboleum castaneum* PINK1 readily phosphorylates the PINKtide peptide and Histone H1 *in vitro*, neither of these non-physiological substrates is phosphorylated by human PINK1. Nonetheless, both Tc and human PINK1 phosphorylate Parkin and Ubiquitin, two physiological substrates of PINK1. Our results show that the substrate selectivity differs among PINK1 orthologues, an important consideration that should be taken into account when extrapolating findings back to human PINK1.

## Introduction

Parkinson’s disease (PD) is the most common neurodegenerative movement disorder, affecting over 1% of the elderly population [1]. While the majority of cases is sporadic, mutations in several genes cause familial PD as reviewed in [2]. One of these genes, *PINK1*, encodes a kinase with a mitochondrial targeting sequence [3]. PINK1 regulates mitochondrial biology through a wide array of downstream targets [4]. However, due to the lack of reliable assays to measure PINK1 kinase activity, it remains mostly unclear whether these targets are direct or indirect substrates of PINK1. Purification of active recombinant human PINK1 has been a technical challenge and only a limited amount of studies report the activity of human PINK1, often using a truncated version of the kinase [5–7].

The discovery that several insect orthologues of PINK1 show a much higher catalytic activity *in vitro* when compared to human PINK1 signified an important step forward in the PINK1 field [8]. Many groups have since employed *Triboleum castaneum* (Tc) PINK1 to address questions on *in vitro* kinase activity and substrate identification or confirmation, especially with regard to the phosphorylation of Ubiquitin and the E3 Ubiquitin ligase Parkin in the context of mitophagy [9–13]. A peptide library screen also led to the identification of the PINKtide, a peptide that is highly phosphorylated by TcPINK1 and provides insight into the sequence preference of the kinase [8]. Several other non-physiological substrates have been used to study the catalytic activity of Tc or human PINK1 *in vitro*, including α-casein, Histone H1, and myelin basic protein [5,8,14].

Nevertheless, it remains unclear why the exotic TcPINK1 orthologue is remarkably more active than human PINK1. Since there are almost no comparative studies between the different orthologues, it is difficult to interpret and compare the results of the reports on substrate phosphorylation using human PINK1 and using TcPINK1. Moreover, PINK1’s controversial localization fuels the discussion about which candidate substrates are physiologically relevant. As these candidates are localized in the inner mitochondrial membrane (NDUFA10 [15]), the intermembrane space (TRAP1 [16] and HtrA2 [17]), the outer mitochondrial membrane (Miro [18], Mfn2 [19], and Bcl-xL [20]), and the cytosol (Parkin [12] and Ubiquitin [9–11]) the interpretation of their ability to be directly phosphorylated is crucial to understand the functional role of PINK1 trafficking and processing. Thus, the implications of potential differences between human and TcPINK1 are not merely technical but can provide insight into the biological role of PINK1 in health and disease.

We have previously reported the set up of a robust *in vitro* radioactive kinase assay for full-length human PINK1 and found that N-terminal processing of human PINK1 affects its autophosphorylation activity [21]. Based on the importance of processing for human PINK1, we hypothesize that the structural differences between human and TcPINK1 would also affect the kinase activity. Here, we compare the activity and substrate preference of human PINK1 to that of TcPINK1. Our results show that both orthologues are active under different *in vitro* conditions and also show differences in substrate specificity.

## Materials and Methods

### Plasmids

TcPINK1 constructs were kindly provided by Miratul Muqit, University of Dundee [8]. Full-length cDNA of human PINK1 and mouse Parkin was obtained from Origene and cloned into pcDNA3.1-V5/His-TOPO according to the manufacturer’s protocol (Life Technologies). A 3xFLAG/Strep-tag was amplified via PCR [22] and cloned on the C-terminus of *PINK1* in the pcDNA3.1 vector using conventional cloning methods. Briefly, PCR products as well as digested vectors were run on 1.5 and 0.8% agarose gels, respectively, and extracted using the Qiaquick Gel Extraction kit (Qiagen). Ligation was performed using T4 DNA ligase (Promega) according to a 1 to 3 molar ratio of vector and insert.

To obtain kinase inactive (KI) human PINK1, Lysine 219 in the ATP-binding pocket and Aspartic acid 362 in the catalytic core were both mutated to Alanine (K219A D362A) by QuickChange II XL site-directed mutagenesis (Agilent technologies) according to the manufacturer’s guidelines. After PCR amplification, the parental non-mutated DNA is specifically digested and the remaining mutated plasmid DNA is transformed. Subsequently, the DNA of single colonies is analyzed for the presence of the mutation via restriction digest where possible, and Sanger sequencing.

The Ubiquitin-like (Ubl) domain of Parkin was obtained by PCR amplification of amino acids 1 to 108 from the pCMV-Parkin plasmid (Origene). The PCR product was further cloned into pGEX-4T-1 (Addgene) in frame with the N-terminal GST-tagged fusion construct. The successful cloning of all plasmids was confirmed by performing sequencing analysis.

### *Triboleum castaneum* PINK1 purification and *in vitro* kinase assay

MBP-tagged *Triboleum castaneum* (Tc) PINK1 was expressed in BL21 bacteria and purified as previously described [8]. Bacterial pellets were lyzed in lysis buffer containing 50 mM Tris-HCl pH 7.5, 150 mM NaCl, 1% Triton X-100, 2 mM EDTA, 0.1% β-mercaptoethanol, 0.2 mM PMSF, and 1 mM benzamidine. Cleared lysate was added to amylose resin for binding at 4°C for 2 h. After several washes with wash buffer (50 mM Tris-HCl pH 7.5, 150 mM NaCl, 0.1 mM EGTA, 0.03% Brij-35, 0.1% β-mercaptoethanol, 0.2 mM PMSF, and 1 mM benzamidine), MBP-TcPINK1 was eluted with 12 mM maltose. The eluted fractions were dialyzed overnight at 4°C in 50 mM Tris-HCl pH 7.5, 150 mM NaCl, 0.1 mM EGTA, 0.03% Brij-35, 0.7% β-mercaptoethanol and 270 mM sucrose and subsequently snap-frozen.

To measure peptide substrate phosphorylation, assays were set up in a total volume of 50 μL, using 1 μM kinase and 1 mM of the peptide substrates. For protein substrates, 1 μg of kinase and 2 μM substrate were incubated in a total volume of 40 μL. In both cases the assay was performed in 50 mM Tris–HCl (pH 7.5), 0.1 mM EGTA, 10 mM MgCl_2_, 2 mM dithiothreitol (DTT) using 100 μM [γ-^32^P]-ATP (Perkin Elmer). Assays were incubated for 30 min at 30°C while shaking at 1200 rpm.

Peptide phosphorylation reactions were terminated by spotting the reaction mixture onto P81 phosphocellulose paper and three washes with 50 mM phosphoric acid for 10 min. After a final wash in 100% acetone, incorporation of [γ-^32^P]-ATP was quantified by scintillation counting. Kinase activity was expressed in units per mg, as previously described (Hastie et al. 2006), where 1 unit represents the incorporation of 1 nmol phosphate into the peptide or protein substrate per 1 min.

Protein substrate reactions were terminated by the addition of LDS sample buffer (Life Technologies) with 4% β-mercaptoethanol. The samples were separated by SDS-PAGE electrophoresis and transferred onto a PVDF membrane. Incorporation of radiolabelled phosphor was assessed via a storage phosphor screen and development on a Typhoon FLA-7000.

### Human PINK1 purification and *in vitro* kinase assay

The procedure for human PINK1 purification and kinase activity measurement was adapted from Hertz et al. [7] and previously described in Aerts et al. [21]. Briefly, COS cells were transfected with pcDNA-3.1-hPINK1-3xFLAG/Strep using TransIT transfection reagent (Mirus Bio) according to the manufacturer’s instructions. Forty-eight hours post transfection, cells were harvested and lyzed in 25 mM Tris-HCl pH 7.5, 150 mM NaCl, 5 mM NaF, 1 mM MgCl_2_, 1 mM MnCl_2_, 0.5% Igepal, mammalian protease inhibitors (Sigma), Complete protease inhibitor (Roche), 50 mg/L DNAse I, 50 mg/L RNAse A, and 1 mM DTT, and homogenized using a 22-G needle in 5 strokes. After 25 min of centrifugation at 20,000× g, the cleared lysate was incubated with FLAG-magnetic beads (Sigma) at 4°C for 45 min. The unbound fraction was separated by magnetic force and removed, and the beads were washed 2 times in lysis buffer, followed by 3 washes with kinase assay buffer (50 mM Tris-HCl pH 7.5, 150 mM NaCl, 10 mM MgCl_2_, 3 mM MnCl_2_, and 0.5 mM DTT).

Kinase assays were performed immediately after purification, with hPINK1-FLAG bound on the beads and 1 to 2 μg of recombinant substrate protein and 100 μM ATP containing 5 μCi [γ-^32^P]-ATP in kinase assay buffer containing 10 mM DTT. Reactions were incubated at 22°C while shaking at 14,000 rpm for 1 h.

For peptide phosphorylation reactions, the supernatant fraction was separated by magnetic force and spotted onto P81 phosphocellulose paper squares. These subsequently underwent three 10-min washes in 50 mM phosphoric acid, and after a final wash in 100% acetone, incorporation of [γ-^32^P]-ATP was quantified by scintillation counting. PINK1 was eluted from the beads by incubation in NuPage LDS sample buffer (Life Technologies) with 4% β-mercaptoethanol at 70°C for 10 min and vortexing twice.

Protein substrate assay samples and eluted PINK1 were separated by SDS-PAGE electrophoresis and transferred onto PVDF membrane. Incorporation of radiolabelled phosphor was assessed via a storage phosphor screen and development on Typhoon a FLA-7000. The membranes were afterwards subjected to western blotting for the detection of PINK1 (FLAG) and the substrates.

### Substrate expression and purification

BL21 bacteria were transformed with N-terminally GST-tagged Ubl domain of Parkin in pGEX-4T-1. For GST-Ubl Parkin, protein expression was induced using 100 μM IPTG, and cells were incubated at 280 rpm and 37°C for 2 h. Bacterial pellets were lyzed in 50 mM Tris-HCl pH 7.5, 150 mM NaCl, 1% Triton X-100, 2 mM EDTA, 0.1% b-mercaptoethanol, 0.2 mM PMSF and 1 mM benzamidine and purified using Glutathione SepharoseTM 4B (GE Healthcare) according to the manufacturer’s instructions.

PINKtide (KKWI(pY)RRSPRRR) peptide was synthesized by Biomatik, based on the sequence provided in Woodroof et al. [8]. Purified Ubiquitin-His (Sigma) and Histone H1 (Merck) were commercially obtained. The quality and purity of all substrates was assessed via SDS-PAGE followed by Coomassie staining.

## Results

**Fig 1:**
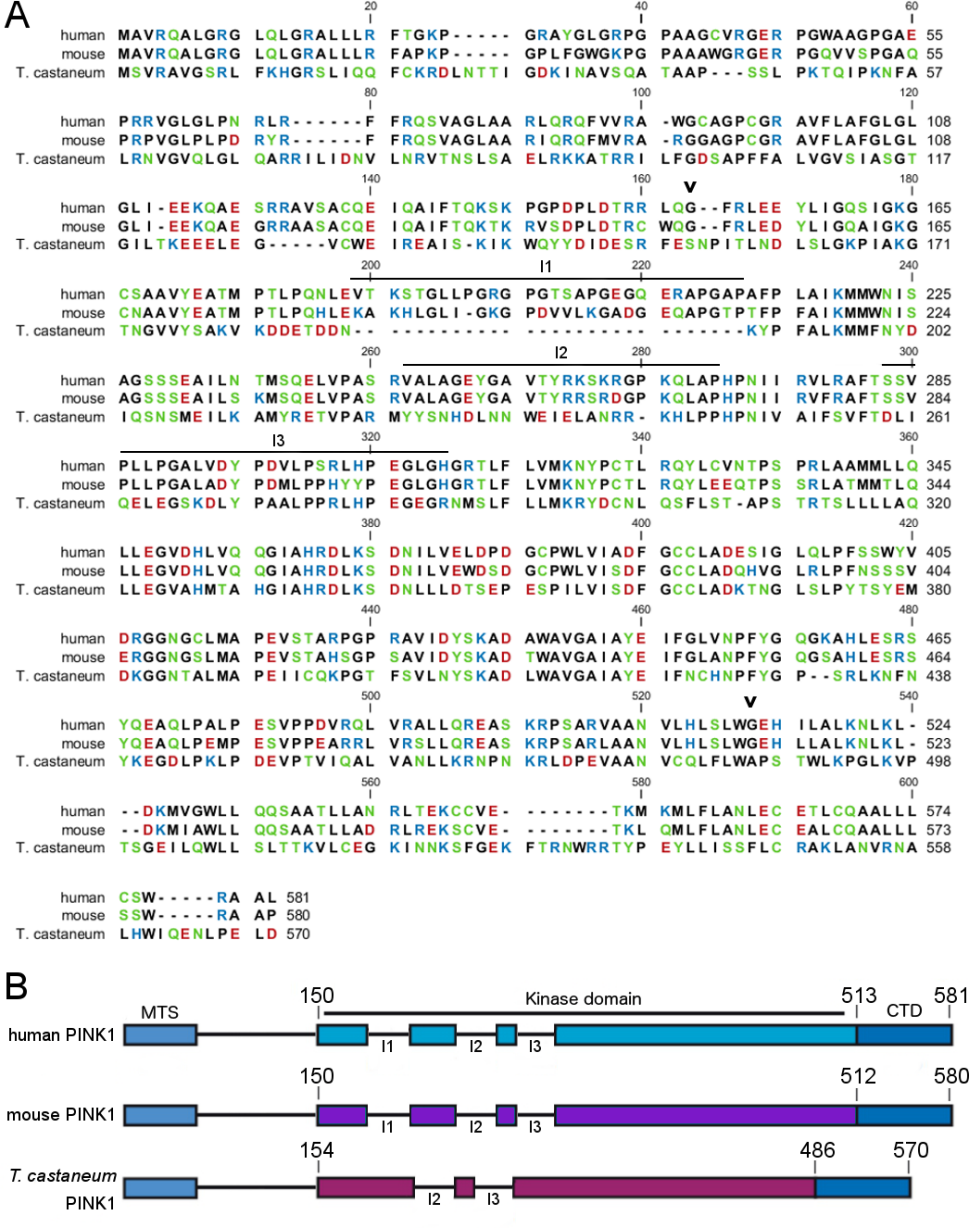
Human, mouse and *T*. *castaneum* PINK1 alignment. (A) Sequence alignment of human, mouse, and *T*. *castaneum* PINK1. Mouse and human PINK1 are 82% identical; Tc and human PINK1 are 36% identical. Polar, non-charged amino acids (green), basic (blue), and negatively charged hydrophilic (red) amino acids are indicated, as well as the start and end of the kinase domain (black arrowhead), and the 3 unique insertions (I1, I2, and I3). (B) Schematic representation of human, mouse, and Tc PINK1 indicating one of the 3 unique insertion regions in the kinase domain of PINK1 is lacking in TcPINK1 (MTS, mitochondrial targeting sequence; CTD, C-terminal domain).

Sequence alignment of both orthologues shows that human and Tc PINK1 are 36% identical (Fig 1A). The respective kinase domains are 41.2% identical. The most important difference is that out of the 3 unique insertion loops in the kinase domain of human PINK1, only two are present in TcPINK1 (Fig 1B) [8,23]. Based on their spatial arrangement, these insertion loops have been proposed to act as docking sites for regulatory proteins [23], however, their function has not been experimentally analyzed.

**Fig 2:**
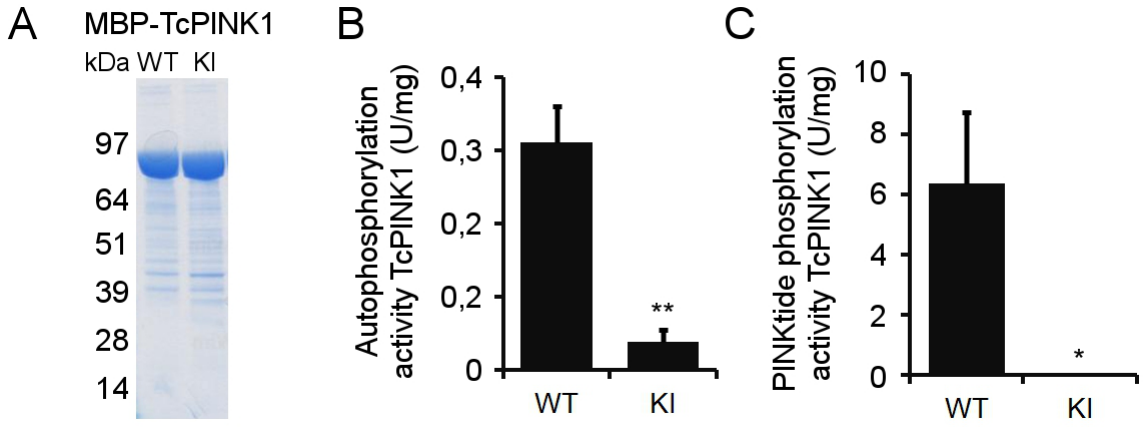
TcPINK1 autophosphorylates and phosphorylates PINKtide. (A) Purity of *E*. *coli*-expressed WT and KI TcPINK1 was evaluated by Coomassie staining. Both forms of TcPINK1 are equally enriched. (B) Quantification of [γ-32P]-ATP *in vitro* autophosphorylation of purified WT or KI TcPINK1. (C) Quantification of [γ-32P]-ATP *in vitro* phosphorylation of PINKtide by purified WT or KI TcPINK1 (mean ± SEM, n = 4 independent experiments). Statistical significance was calculated between WT and KI TcPINK1 using Student’s *t*-test (*: p-value < 0.05; **: p-value < 0.01).

We assessed the *in vitro* kinase activity of both recombinant Tc and human PINK1. For this, we expressed TcPINK1 with an N-terminal maltose binding protein (MBP) tag in *E*. *coli* [8]. Fusion with MBP dramatically enhances the solubility of proteins otherwise accumulating in the insoluble fraction [24], and allows for purification of the fusion protein on an amylose resin under non-denaturing conditions. Coomassie staining confirmed successful purification of both wild type (WT) and kinase inactive (KI) MBP-tagged TcPINK1 (Fig 2A). The KI TcPINK1 is catalytically inactivated by mutation of D359, one of the conserved Aspartic acids in the catalytic core, corresponding to D384 in hPINK1 [8]. Using both forms of TcPINK1, we set up an *in vitro* kinase assay with radiolabelled ATP, with and without PINKtide substrate. The measurement of radiolabelled phosphor incorporation through scintillation counts showed that WT TcPINK1 is catalytically active. We detect PINK1 autophosphorylation as well as high levels of PINKtide phosphorylation specifically for WT, and not for KI TcPINK1 (Fig 2B and 2C), consistent with previous reports [8,12].

**Fig 3:**
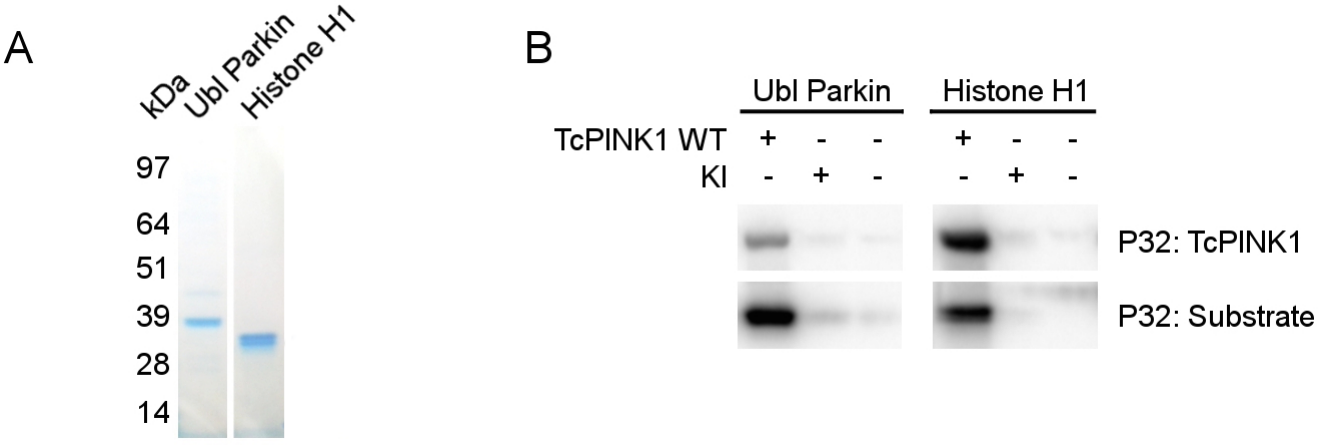
TcPINK1 phosphorylates Histone H1 and the Ubl domain of Parkin. (A) Purity of Histone H1 and immunoprecipitated GST-tagged Ubl Parkin was evaluated by Coomassie staining. (B) *In vitro* phosphorylation assay using [γ-32P]-ATP, purified TcPINK1, and Ubl Parkin or Histone H1 shows that both are specifically phosphorylated by WT and not KI TcPINK1. WT TcPINK1 also displays autophosphorylation activity.

Since WT TcPINK1 is active *in vitro*, we proceeded to test whether it can directly phosphorylate several putative PINK1 substrates. We successfully purified the recombinant GST-tagged Ubl domain of Parkin [12], and obtained commercially available purified Histone H1, a non-physiological PINK1 substrate (Fig 3A) [5]. In addition to the PINKtide, TcPINK1 is able to directly phosphorylate both of these proteins *in vitro* (Fig 3B).

Next, we tested the activity of human PINK1 towards these TcPINK1 substrates. We used a 3xFLAG tag to purify human PINK1 after expression in mammalian cells. The 3xFLAG tag is a convenient epitope for immunoprecipitation that, like MBP, allows for elution in non-denaturing conditions. However, we obtained very low elution recoveries for PINK1, most probably due to PINK1 protein aggregation, and therefore, we proceeded by conducting *in vitro* kinase assays with PINK1 still bound to anti-FLAGM2 beads. When using the same *in vitro* assay set up as we used for TcPINK1, we could not detect kinase activity for human PINK1 (data not shown). However, as we have previously reported [21], we succeeded in obtaining active human PINK1 by modifying both the purification and assay conditions. Crucially, we shortened the overall time frame of protein purification, allowing less than 2 h between cell lysis and the kinase assay, which was performed at 22°C instead of 30°C. We further verified whether addition of detergent, BSA, reducing agents, or protease and phosphatase inhibitors in the kinase assay buffer could increase the detected phosphorylation and found a marked increase in catalytic activity in highly reducing conditions using 10 mM or more DTT (S1 Fig).

**Fig 4:**
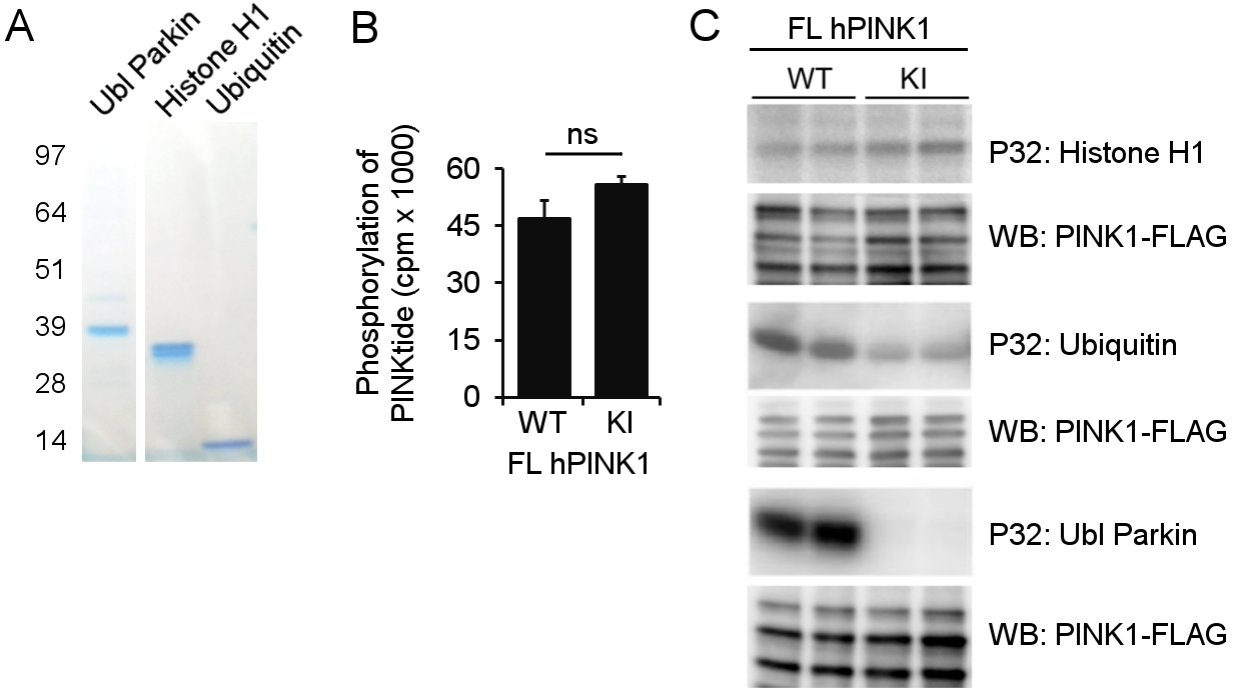
Human PINK1 phosphorylates Parkin and Ubiquitin, but not PINKtide and Histone H1 *in vitro*. (A) Coomassie staining of the different substrates tested for *in vitro* phosphorylation by human PINK1. (B) Quantification of *in vitro* [γ-32P]-ATP PINKtide phosphorylation by purified human PINK1-FLAG. Human PINK1 did not specifically phosphorylate the PINKtide *in vitro* (n = 2 technical replicates, cpm: counts per minute). (C) An *in vitro* phosphorylation assay using [γ-32P]-ATP was performed with purified WT and KI human PINK1-FLAG and different putative PINK1 substrates. While WT PINK1 specifically phosphorylates both Parkin and Ubiquitin, Histone H1 was not found to be phosphorylated *in vitro*. Anti-FLAG WB shows equal loading of WT and KI human PINK1 (the full-length and 2 processed PINK1 forms are shown; note that the samples for Ubiquitin were run on a different gel type, causing a different migration pattern for PINK1).

Using this set-up we previously reported that only a truncated form and not the full-length form of human PINK1 undergoes autophosphorylation *in vitro*, and this occurs independent of prior mitochondrial depolarization [21]. In addition we show specific phosphorylation of both the Ubl domain of Parkin and Ubiquitin using full-length human PINK1. To be able to compare human PINK1 to TcPINK1, we further tested the direct phosphorylation of Histone H1 and PINKtide peptide. We found that neither of them was phosphorylated by full-length human PINK1 (Fig 4).

In summary, our data show that while both Tc and human PINK1 are catalytically active *in vitro*, their kinase activity is regulated differently. Not only are different experimental conditions required to maintain both orthologues in an active conformation, they also show differences with regards to substrate selection and phosphorylation, as not all substrates phosphorylated by TcPINK1 are phosphorylated by human PINK1.

## Discussion

Our work reveals that although both Tc and human PINK1 are active *in vitro*, human PINK1 requires more stringent purification and activity measurement conditions to remain in its active conformation. The difficulties reported by other researchers in the field undertaking similar efforts corroborate this notion [5,8,17]. Essential factors for the human PINK1 *in vitro* phosphorylation assay are time and temperature. Although kinase assays are usually performed at 30°C, we observe specific human PINK1 kinase activity by incubating PINK1 at 22°C for 1 h. The reasons for obtaining better results at this low temperature are unclear. Delayed activity measurement also strongly reduces the resulting phosphorylation signals. While loss of enzymatic activity over time is not necessarily unexpected, it remains unclear why such a rapid loss of PINK1 kinase activity is observed. Additionally, strong reducing conditions stimulate PINK1 catalytic activity, as an increased phosphorylation signal was observed with 10 mM DTT. The use of such high DTT concentrations in kinase assays is uncommon, but can stimulate activity through conformational changes as is the case for insulin receptor kinase [25,26].

Because radioactive read-outs allow for the direct measurement of enzyme activity in a highly sensitive and accurate manner, they are ideally suited for the study of enzyme kinetics and for the confirmation of direct substrates. Our results confirm that Parkin is a direct substrate of TcPINK1 (Fig 3). We also show that both Parkin and Ubiquitin are phosphorylated by human PINK1 (Fig 4). However, as with every method, there are some limitations. One is that an active kinase can phosphorylate non-physiological targets *in vitro*. Nevertheless, such non-physiological targets can be used to study enzyme kinetics. In the case of PINK1 for example, the non-physiological substrate α-casein has been used to study the effects of different mutations on kinase activity of PINK1 [14]. We show that although the non-physiological substrates Histone H1 and PINKtide peptide prove useful to measure TcPINK1 activity *in vitro*, they are not specifically phosphorylated by human PINK1.

Kinase substrate recognition can occur through a consensus phosphorylation sequence on the substrate, or through distal interactions mediated by docking motifs spatially separated from the phosphorylation site in the substrate and the active site of the kinase [27]. Discrepancies between the two kinases could thus be the result of both proximal and distal kinase-substrate interactions. Bioinformatic analysis predicts that the two orthologues exhibit different target sequence preferences [23]. The fact that the PINKtide is not specifically phosphorylated by human PINK1 demonstrates that the consensus phosphorylation sequence of both kinases is indeed different and undermines the value of peptide library screens using insect orthologues instead of the human protein. However, differences in Histone H1 phosphorylation are most likely due to altered distal interaction parameters, possibly caused by the absence of one of the unique insertion loops in the N-terminal lobe of the PINK1 kinase domain (Fig 1).

Although TcPINK1 is a useful tool for the study of PINK1 activity, we warrant cautious interpretation of substrate validation using non-human orthologues of PINK1. We present a proof-of-concept study on the differences in activity of insect and human PINK1 using a select but widely used substrate set. Further research on other putative PINK1 substrates, including HtrA2 [17], TRAP1 [16], Mitofusin 2 [19], Miro [18], NDUFA10 [15], and Bcl-xL [20], is required to unravel the relevance and impact of these differences in activity and of the respective candidate substrates. We propose that PINK1 kinase activity is regulated not only by phosphorylation [12,21,28,29], but possibly also by structural alterations [4,21]. It is well established that PINK1 is processed by several mitochondrial proteases, and we speculate that this could have important effects on substrate phosphorylation. Taking the previously reported differences in autophosphorylation for full-length and truncated human PINK1 into account [21], these data underscore the importance of a more thorough understanding of the structure-function relation of the PINK1 kinase in both physiological and pathological conditions.

## Supporting Information

S1 Fig Optimization of assay conditions for human PINK1. (A) *In vitro* phosphorylation assays terminated after 10, 30, or 60 min, using [γ-^32^P]-ATP and purified human PINK1 and Ubiquitin show that decreasing the assay time does not lead to a decrease in unspecific activity. (B) *In vitro* phosphorylation assays using [γ-^32^P]-ATP and purified human PINK1 and Ubiquitin, incubated at either 22°C or 30°C, demonstrate that specific PINK1 activity can only be detected at a decreased temperature. (C) *In vitro* phosphorylation activity of human PINK1 towards both Parkin and Ubiquitin is higher in 10 mM DTT.
(TIF)
