# Supplementary figures and images for: In Vitro Comparison of the Activity Requirements and Substrate Specificity of Human and Triboleum castaneum PINK1 Orthologues
Source: PLoS One. 2016 Jan 19;11(1):e0146083. doi: 10.1371/journal.pone.0146083 (PMC4718624; doi:10.1371/journal.pone.0146083)

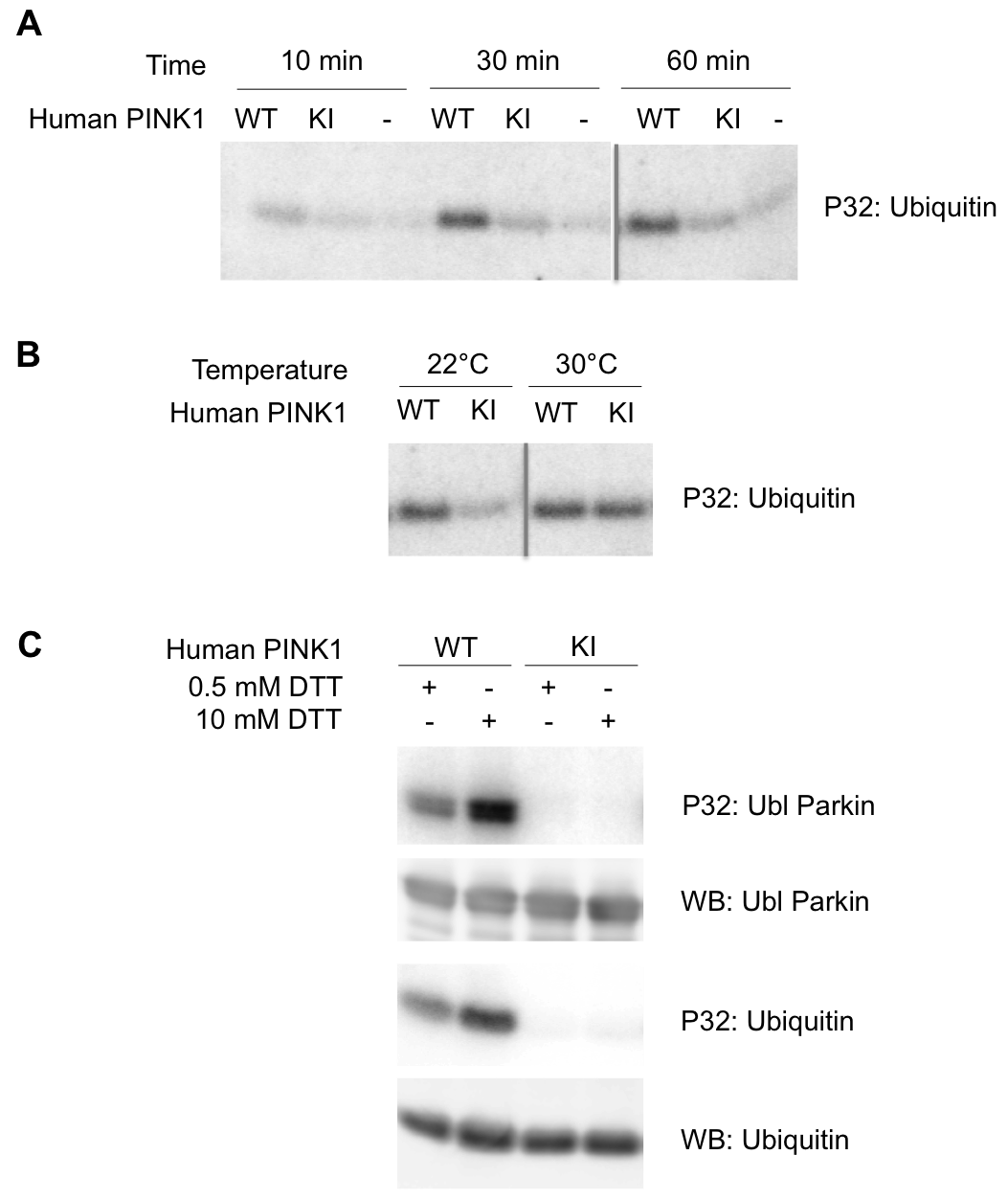

Supplement: S1 Fig — (A) In vitro phosphorylation assays terminated after 10, 30, or 60 min, using [γ-32P]-ATP and purified human PINK1 and Ubiquitin show that decreasing the assay time does not lead to a decrease in unspecific activity. (B) In vitro phosphorylation assays using [γ-32P]-ATP and purified human PINK1 and Ubiquitin, incubated at either 22°C or 30°C, demonstrate that specific PINK1 activity can only be detected at a decreased temperature. (C) In vitro phosphorylation activity of human PINK1 towards both Parkin and Ubiquitin is higher in 10 mM DTT. (TIF) [file pone.0146083.s001.tif]
